# Development and Types of Passive Samplers for Monitoring Atmospheric NO2 and NH3 Concentrations

**DOI:** 10.1100/tsw.2001.82

**Published:** 2001-10-09

**Authors:** Y.S. Tang, J.N. Cape, M.A. Sutton

**Affiliations:** Centre for Ecology and Hydrology, Edinburgh Research Station, Bush Estate, Penicuik, Midlothian EH26 0QB, UK

**Keywords:** passive diffusion sampling, diffusion tube, badge samplers, monitoring methods, nitrogen dioxide (NO_2_), ammonia (NH_3_)

## Abstract

Numerous passive samplers based on the ‘Palmes-tube’ have been developed for ambient air monitoring. In each case, the diffusion path length and/or crosssectional area are modified to achieve the desired sampling rate. ‘Tube-type’ samplers are low sensitivity samplers suitable for long-term monitoring, whereas the ‘badge-type’ samplers have faster sampling rates suited to short-term monitoring. In the U.K., diffusion tubes are widely used for monitoring nitrogen dioxide (NO2) and ammonia (NH3). The open-ended diffusion tubes are prone to positive bias caused by incursion of wind eddies, leading to a shortening of the diffusion path. By using a porous membrane at the inlet, wind incursion is prevented, but an additional diffusion resistance is imposed and it is necessary to calibrate the tubes against a reference method to obtain an effective sampling rate. For NO2 sampling, positive bias also arises from the reaction of NO with O3 within the sampler. The interference from the chemical reaction is severe close to NO sources, with errors up to 30% for curbside locations when using the ‘tubetype’ sampler. In rural areas, where NO concentrations are small relative to NO2, these errors are small. In some implementations, there is also a negative bias over long sampling periods caused by the degradation of trapped NO2. The low sampling rates of diffusion tubes make them too uncertain for use at background NH3 concentrations (<1 μg NH3 m-3) where they significantly overestimate concentrations. Badge-type samplers such as the ‘Willems badge’ samplers permit accurate sampling at low ambient NH3 concentrations, but suffer from saturation at high concentrations and sensitivity to wind speed. A passive sampler optimised for monthly measurements of NH3 is reported here, together with its application in the U.K. National Ammonia Monitoring Network.

### TABLE OF CONTENTS

Introduction
Passive Diffusion Sampling
    Principles of Passive Diffusion Sampling
    Types of Passive Diffusion Samplers
Effects of Wind Speed on Passive Sampling
Passive Sampling of $NO_2$
    Effects of Within-Tube Chemical Reaction on Passive Sampling for $NO_2$
    Effects of Exposure Time on Passive Sampling for $NO_2$
Passive Sampling of $NH_3$
    Uncertainties in the Reported Application of $NH_3$ Diffusion Tubes
    Performance of Badge Samplers for $NH_3$
    Contamination / Operator-Specific Error
Discussion
    Recommendations for $NO_2$ and $NH_3$ Sampling
    Application of a Modified Passive Sampler for $NH_3$
Conclusions
Acknowledgements
References

### INTRODUCTION

Nitrogen dioxide ($NO_2$) and ammonia ($NH_3$) are important trace gas pollutants in the atmosphere, which can acidify terrestrial ecosystems following dry deposition. Their reaction products can also contribute to wet deposited acidity, whereas the formation of aerosols can impact visibility and influence the transport characteristics of these gases[1]. For $NO_2$, the main concern is adverse effects on human health through the formation of photochemical oxidants. Within the U.K., local authorities have a statutory requirement to monitor $NO_2$ concentrations, where an annual mean standard of 21 ppb $NO_2$ has been introduced within the U.K. National Air Quality Strategy[2]. Deposition of atmospheric $NH_3$ to ecosystems is also known to cause eutrophication effects[3,4], and the measurement of $NH_3$ concentration is important in allowing estimates of atmospheric N deposition to ecosystems to be made.

Passive diffusion samplers are widely used in the monitoring of both $NO_2$ and $NH_3$. Compared with conventional active monitoring methods (e.g., chemiluminescence analyzer for $NO_2$, active denuder method for $NH_3$), they have many clear advantages. These advantages include low cost and no requirement for power supply, in addition to simplicity and flexibility of deployment and use. They can be used virtually anywhere and large numbers of them can be deployed to provide detailed spatial and temporal surveys. Specialist training and maintenance are also not required to deploy them in the field, further reducing the operational costs of monitoring work with these samplers.

To obtain accurate and reliable monitoring data from passive diffusion samplers, an understanding of the operating principles of diffusion sampling, in conjunction with knowledge of the factors that may affect sampler performance, are essential.

## PASSIVE DIFFUSION SAMPLING

### Principles of Passive Diffusion Sampling

All passive samplers operate on the principle of diffusion of gases from the atmosphere along a sampler of defined dimensions onto an absorbing medium, according to Fick's law. The theoretical uptake rate of a sampler is a function of the length, $L$ (m), and the cross-sectional area, $A$ ($m^2$), of the stationary air layer within the sampler, and can be calculated provided that the diffusion coefficient, $D$ ($m^2\ s^{-1}$), of the gas of interest is known. The diffusion path length is nominally the distance from the mouth of the sampler to the reaction surface at the other end of the tube. Disturbance to the stationary air layer, such as through the effect of wind turbulence, can shorten the nominal path length (see reviews[5,6]). Conversely, the use of a turbulence-damping membrane at the inlet can increase the effective path length due to a quasi-laminar layer of air forming adjacent to the outside of the membrane[7].

The effective volume of air sampled ($V$, $m^3$) is given by:

$$V = DAt/L \tag{1}$$

where $t$ = time of exposure(s). For $NO_2$, $D = 0.154 \times 10^{-4}\ m^2\ s^{-1}$ at 20°C[8], while for $NH_3$, $D = 2.09 \times 10^{-5}\ m^2\ s^{-1}$ at 20°C[9].

The air concentration of a pollutant ($\chi$, e.g., $\mu g\ m^{-3}$) can then be calculated as:

$$\chi = (m_e - m_b) / V \tag{2}$$

where $m_e$ = amount of a pollutant collected on an exposed sample (e.g., µg) and $m_b$ = amount of a pollutant in the blank sample (e.g., µg).

### Types of Passive Diffusion Samplers

Numerous samplers based on the original 'Palmes-tube'[8] have been developed for ambient air monitoring. In each case, the diffusion path length and/or cross-sectional areas are modified to achieve the desired sampling rate. In particular, tube- and badge-type samplers are used extensively to measure atmospheric $NO_2$ and $NH_3$. A summary of passive samplers described in the literature for these pollutants is given in Table 1.

The tube-type samplers are usually hollow cylindrical tubes oriented vertically (Fig. 1). A cap at the top end holds either a filter paper or stainless steel grid in place, which is coated with an absorbent that collects the gas of interest. For $NO_2$ sampling, the most commonly used absorbent is triethanolamine (TEA)[8]. For $NH_3$, a number of acids have been used, including citric, phosphoric, sulphuric, and tartaric[20]. However, oxalic acid should be avoided because it is volatile and loss of the oxalic acid coating can occur during sampling. The lower end of the tube is either left open as in the case of the longer 7.1-cm open diffusion tube[22] or capped with a membrane in the case of the 3.5-cm membrane diffusion tube[12] and the badge-type samplers (Fig. 1)[7,15].

## EFFECTS OF WIND SPEED ON PASSIVE SAMPLING

In windy conditions, air moving over the open end of a diffusion tube generates turbulence inside that can lead to a reduction in the diffusion length, referred to as 'wind-shortening'[6,9,11]. This results in an increase in the effective sampling rate so that the air concentration is overestimated if the theoretical rate is used. The increase is variable, depending on wind speed and location of the samplers[15,23], because the angle of wind is also important[10]. Fig. 2 shows the relationship between overestimation of $NO_2$ concentration with increasing wind speed when open diffusion tubes are used from a number of controlled experiments in a wind tunnel[10].

### TABLE 1
## Examples of Passive Diffusion Samplers Used in Monitoring of Atmospheric $NO_2$ and $NH_3$ Concentrations

| Type | Dimensions L(cm)xA(cm$^3$) | Inlet | Collection surface | Collection medium – $NO_2$ | Collection medium - $NH_3$ | $NO_2$ Uptake Rate (m$^3$ h$^{-1}$)* | $NH_3$ Uptake Rate (m$^3$ h$^{-1}$)* | Comment / Reference |
|---|---|---|---|---|---|---|---|---|
| Palmes-type Diffusion Tube | 7.1 x 0.95 | Open | Stainless steel grids | Triethanolamine | Sulphuric acid | $7.17 \times 10^{-5}$ | $9.74 \times 10^{-5}$ | Protective cover recommended for outdoor sampling. [8,10,11,22] |
| Palmes-type Diffusion Tube | 3.5 x 0.95 | Membrane | Stainless steel grids | Not reported | Sulphuric acid | $1.50 \times 10^{-4}$ | $2.03 \times 10^{-4}$ | Not tested for $NO_2$. Protective cover used for outdoor sampling. [12, 13] |
| Blatter Diffusion Tube | 0.7 x 0.64 | Open | Absorbent behind Teflon membrane | Not reported | Ethylene glycol / HCl | $5.04 \times 10^{-3}$ | $6.84 \times 10^{-3}$ | Protective cover used for outdoor sampling. [14] |
| Ferm Badge | 1.0 x 3.14 | Steel grid + Teflon membrane | Whatman 40 | KI / $NaAsO_2$ NaI / NaOH | Citric acid | $1.74 \times 10^{-3}$ | $2.36 \times 10^{-3}$ | [15] |
| Willems Badge | 0.2 x 5.31 | PTFE membrane | Glass fibre filter | NaI / NaOH | Tartaric acid | $1.47 \times 10^{-2}$ | $2.00 \times 10^{-2}$ | Additional 0.8 cm inlet in front of membrane. [7] |
| CEH ALPHA | 0.6 x 3.46 | PTFE membrane | Schleicher & Schuell 595 | Under test | Citric acid | $3.20 \times 10^{-3}$ | $4.34 \times 10^{-3}$ | Protective cover used for outdoor sampling. |
| Ogawa Badge | 0.6 x 0.79 (25 holes of 0.2 cm diameter) | Open | Stainless steel grid + Filter Pad | Triethanolamine | - | $7.26 \times 10^{-4}$ | $9.85 \times 10^{-4}$ | Not tested for $NH_3$. Protective cover used for outdoor sampling. [16] |
| CSPSS Badge | 8.0 x 17.35 | PTFE membrane | Absorbent behind PTFE membrane | CHEMIX™ | - | $1.20 \times 10^{-2}$ | $1.63 \times 10^{-2}$ | Not tested for $NH_3$ Protective cover used for outdoor sampling. [17] |
| Krochmal Badge | 1.0 x 4.91 | Polypropylene membrane | Whatman 1 or Stainless steel grids | Triethanolamine | - | $2.72 \times 10^{-3}$ | $3.69 \times 10^{-3}$ | Not tested for $NH_3$. [18] |
| Yanagisawa Badge | - | - | - | Triethanolamine | - | - | - | Not tested for $NH_3$. [19] |
| UC Davis Badge | 1.03 x 8.50 | Zefluor membrane | Whatman 41 | Not reported | Citric acid | $4.58 \times 10^{-3}$ | $6.21 \times 10^{-3}$ | Not tested for $NO_2$. [20] |
| Kasper & Puxbaum | 0.90 x 8.04 | Teflon membrane | Stainless steel grids | Not reported | Phosphoric acid | $4.95 \times 10^{-3}$ | $6.72 \times 10^{-3}$ | Protective cover used for outdoor sampling. [21] |

*Calculated theoretical sampling rate, based on the reported dimensions of the samplers, using the sampling rate equation and diffusion coefficients of $NO_2$ and $NH_3$ detailed in section 2.1.

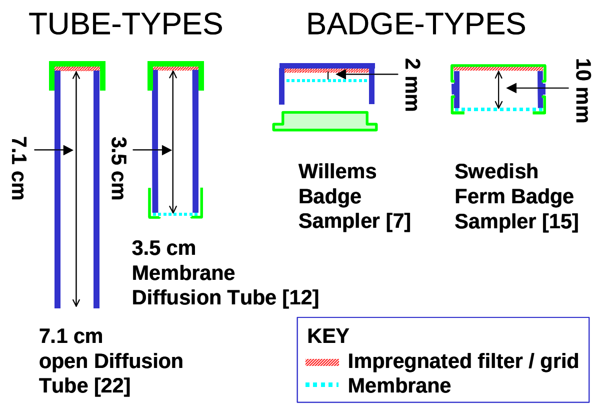

**FIGURE 1.** Some examples of tube-type and badge-type passive diffusion samplers.

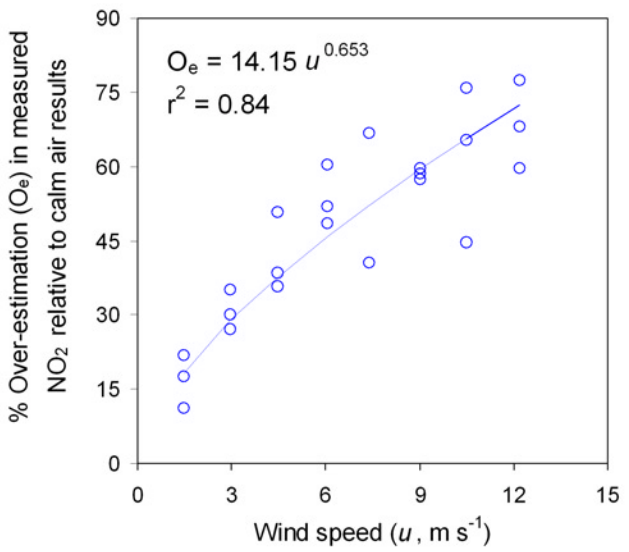

**FIGURE 2.** Effect of wind speed on the over-estimation of $NO_2$ concentration for 71 mm diffusion tubes (adapted from Hargreaves [10]).

An intercomparison study conducted by Campbell et al.[23] showed that the open diffusion tubes overestimated $NO_2$ by as much as 30% when compared against a reference chemiluminescence analyzer. $NO_2$ overestimation varied between different sites, with the largest errors reported at more exposed locations. Their study attributed the $NO_2$ overestimation to the wind-shortening effect on the open-type diffusion tube. This finding is supported by other workers[12,24,25,26]. The wind-shortening effect can be minimised when using open diffusion tubes by the careful selection of sampling sites where vertical movement of air is avoided[6,27], and in exposed windy locations, the use of some form of wind shield can also reduce wind turbulence[6].

Provided that the general guidelines for the location and exposure of the open diffusion tube are followed, wind speed has been considered to have minimal or negligible influence on its sampling rate because of the large length-to-area ratio of the tube[6,27]. For example, a reduction in the path length of 7.1 cm by 1 cm would only result in a 16% overestimation of air concentration, compared with 40% for a tube that is half its length. Atkins and Lee[27] also justified this conclusion on the basis that the largest air concentrations tend to occur in low wind speed conditions. The shorter 3.5-cm diffusion tube and all badge-type samplers have a gas permeable membrane placed at the inlet to establish a turbulent-free layer of air inside the sampler[17,18,26,28,29]. The membrane does, however, impose an additional resistance against gas diffusion, which needs to be taken into account when deriving a sampler's effective sampling rate. This may be achieved by applying a correction factor to the measured concentration using the theoretical rate. This can be determined empirically from field calibration of the samplers against a suitable reference method or from estimates made based on the theoretical resistance of the membrane plus the estimated thickness of the quasi-laminar boundary layer on the outside of the membrane[21], which depends on wind speed and sampler geometry.

Low wind speeds can also be a problem because this can lead to a depletion of the pollutant in front of the sampler. This causes an increase in the depth of the boundary layer ($L_b$) between the membrane and the moving air outside, which increases the effective diffusion length ($L + L_b$) and can result in an underestimation of the air concentration. Badge-type samplers are affected more than tube-type samplers due to a higher ratio of the boundary layer to effective diffusion length ($L_b / (L + L_b)$). The Willems badge shows the greatest sensitivity to low wind speed, since a large layer of stagnant air can also form in front of the membrane within the sampler (length = 8 mm). Willems[7] and Kasper and Puxbaum[21] use an equation derived from the relationship between boundary layer resistance and wind speed to provide corrected estimates of air concentrations.

### PASSIVE SAMPLING OF $NO_2$

In the U.K., open diffusion tubes are routinely used to provide large-scale monitoring and assessment of $NO_2$ to ensure compliance with set Air Quality Objectives[2]. They were used in the Rural $NO_2$ Network between 1987 and 1990[30] and continue to be operated across the U.K. $NO_2$ Tube Network with measurements at over 1,200 sites[31].

There are uncertainties, however, regarding the accuracy and reliability of the open diffusion tubes for air quality assessment. Trials in rural areas have shown good agreement with the European Community (EC) reference method (chemiluminescence analyzer)[22], whereas urban studies have found significant overestimation of $NO_2$ concentrations[23,32,33,34]. Within the U.K. $NO_2$ Network, a field intercomparison exercise carried out in 1998 to test the performance of the $NO_2$ diffusion tubes found that although 79% of the 38 participating laboratories performed to the data quality objectives established in the EU Daughter Directive (±25% compared with the reference chemiluminescence method), the range in the average bias of –39% to +58% was substantial[33]. The preparation technique for the $NO_2$ diffusion tubes was identified as one of the problems[34,40], and the protocol for the preparation of diffusion tubes was subsequently standardised across the network, which recommends the pipetting of a 20% solution of TEA in deionised water directly onto the grids.

Other major potential sampling artefacts associated with the use of the open diffusion tube for $NO_2$ sampling that have been identified include: (1) wind turbulence effects on sampling rate, (2) *in situ* formation of additional $NO_2$ between NO and $O_3$ (ozone) inside sampler during sampling, and (3) exposure-dependent loss of chemisorbed $NO_2$.

#### Effects of Within-Tube Chemical Reaction on Passive Sampling for $NO_2$

The acrylic bodies of open diffusion tubes are effective in blocking most of the UV wavelengths that can photolyse $NO_2$ during daytime[35]. The photolytic pathway that destroys $NO_2$ is therefore blocked, while reaction between NO and $O_3$ molecules diffusing into the tube continues, leading to the formation of 'additional' $NO_2$.

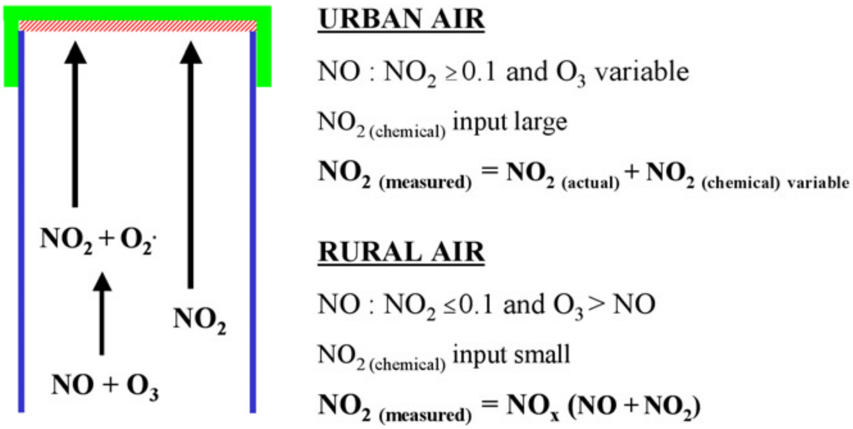

**FIGURE 3:** Sampling artefact due to 'within-tube' chemical reaction leading to formation of 'additional' $NO_2$.

The extent of the chemical interference is governed by the local relative concentrations of NO, $O_3$, and $NO_2$ and most crucially by the proportion of $NO_x$ (= NO + $NO_2$), as shown in Fig. 3. In the urban environment where NO is the predominant species in total $NO_x$, NO measured as $NO_2$ due to chemical reaction can give rise to errors of up to 30% for curbside locations[35], and the errors are likely to be greatest when the $NO_2$:$NO_x$ ratio is about 0.5:1 at the monitoring site[36]. In clean rural air, where NO concentrations are small relative to $NO_2$ and considerably lower than $O_3$, these errors are small and the diffusion tubes will measure total $NO_x$ effectively.

Any attempt to correct the diffusion tube measurements for the chemical overestimation is difficult, since the overestimation of $NO_2$ due to chemistry is dictated by the temporal variations in concentrations of ambient $NO_2$, NO, and $O_3$ during exposure. Close to NO sources, the concentration of NO, $NO_2$, and $O_3$ do not achieve photochemical equilibrium on timescales similar to the sampling rate. To assess the magnitude of the additional formation of $NO_2$, Heal and Cape[32] developed a diffusive transport chemical model to simulate the 'within-tube' chemistry. Predicted $NO_2$ concentrations from the model agreed with measured $NO_2$ concentrations in two independent studies in Edinburgh[35] and Cambridge[36], lending support to the hypothesis that reduced $NO_2$ photolysis accounted for most of the excess $NO_2$ measured with the passive samplers. Another simplified chemical model based on the model of Heal and Cape[32] has also recently emerged[37] with similar conclusions. This revises the earlier conclusions from another model developed by the same group[22] that the chemical interference effect was negligible. This earlier view was suggested on the basis that the errors were thought to be within the acceptable uncertainty limits of the measurements.

Residence times of NO and $O_3$ during diffusion along the sampler also dictate the extent of 'within-tube' chemistry. For the 7.1-cm diffusion tube, the residence times of NO and $O_3$ are of the order of 2.8 min[35], which gives sufficient time for reaction. The residence times of NO and $O_3$ are considerably shorter in badge-type samplers and $NO_2$ formation would therefore be expected to be negligible. Indeed, good agreement was obtained between $NO_2$ concentrations measured using the Ferm sampler and an active reference method[29]. By increasing the diffusion length of the same badge sampler, Ayers et al.[29] also demonstrated an increase in measured $NO_2$ concentrations with an increase in length of the samplers. Kirby et al.[34] provided confirmation that the positive bias increased with tube length of tube-type samplers. They found that the measured $NO_2$ from diffusion tubes was in the order 120 mm > 71 mm ≥ 55 mm. This is entirely in agreement with chemical bias where codiffusing NO and $O_3$ molecules in longer tubes have more time to react to form excess $NO_2$, and shows the lack of a wind speed effect, which would be expected to operate in the opposite direction.

### Effects of Exposure Time on Passive Sampling for $NO_2$

TEA is commonly used for the collection of $NO_2$ in passive samplers, as it is an effective absorbent for $NO_2$[8,9]. $NO_2$ is converted to nitrite ions after reaction with TEA, with conversion assumed to be 100%[38]. There is, however, a lack of specificity of TEA towards $NO_2$, since it is also sensitive to sulphur dioxide ($SO_2$). Where $SO_2$ concentrations are high, this may lead to a decrease in sampling efficiency, since the basicity of the TEA coating is affected by the collection of both $NO_2$ and $SO_2$. There are various reports that $NO_2$ diffusion tubes exposed for 1 month consistently give lower $NO_2$ concentrations than averaged $NO_2$ concentrations from successive samples exposed for 1- or 2-week periods[26,32,39,40].

The mechanism for this time-dependent loss of $NO_2$ is not known. A reduction in sampling efficiency, loss of chemisorbed $NO_2$ on the TEA through *in situ* photolysis[35], or a combination of both factors could be responsible. Smith et al.[41] identified a clear seasonal effect, with the largest loss in $NO_2$ occurring during the summer months. This would be consistent with increased photodegradation of the TEA-$NO_2$ complex in the warm sunny months.

Some workers use a potassium iodide (KI) and sodium arsenite ($NaAsO_2$) mixture to trap $NO_2$[26,39]. The iodide ions catalyse the reduction of $NO_2$ to nitrite, which is stabilised by the alkaline condition provided by $NaAsO_2$. Where $NO_2$ concentrations are high, oxidation of available arsenite to arsenate can lead to loss of alkalinity of the coating and to the loss of already trapped $NO_2^-$ as $HNO_2$[39]. By substituting the arsenite with NaOH, alkaline conditions can be maintained at the sorbent surface[29,39]. Since $NaAsO_2$ is highly toxic, the use of NaOH is preferable, and $NO_2$ measurements using a sodium iodide (NaI) and NaOH mixture did not show $NO_2$ underestimation for long exposure times when compared to weekly sampling[39]. Recently, Chemix$^{TM}$, a new collection medium developed by Maxxam Analytics[17], was also reported to give good quantitative measures of $NO_2$ with their passive sampling system under all outdoor conditions in Canada. However, the chemical composition of Chemix$^{TM}$ has not been published.

## PASSIVE SAMPLING OF $NH_3$

The measurement of atmospheric $NH_3$ with passive diffusion samplers is notoriously problematic, since it is a difficult gas to sample accurately and contamination is a major issue[10,42]. Positive bias at low ambient $NH_3$ concentrations with diffusion tube sampling is widely reported[1,10,13]. A number of attributable factors include: (1) wind-shortening effect on open diffusion tube, (2) sampling of $NH_4^+$ aerosol, (3) uncertainty due to low sampling rates, and (4) uncertainty with regards to field and laboratory tube blanks. Negative bias at higher ambient $NH_3$ concentrations encountered in the Netherlands has also been reported[28], which was thought to be due to either the increased resistance of the membrane inlet of membrane diffusion tubes or loss of sampling efficiency with time. Badge-type samplers such as the 'Willems badge' samplers perform well at low ambient $NH_3$ concentrations, but can only be used for short-term sampling because the samplers become saturated very quickly[43]. An intercomparison conducted by Kirchner et al.[44] on ten tube- and badge-type samplers identified artefacts due to the effect of temperature on different absorbents used, but was unable to draw further conclusions with regard to the performance or recommendation of any of the samplers.

### Uncertainties in the Reported Application of $NH_3$ Diffusion Tubes

Diffusion tubes for the measurement of atmospheric $NH_3$ concentrations have been used for many years[1,9,10,28], but with very mixed success. Although Hargreaves and Atkins[9] found them to perform satisfactorily in the range 1 to 20 $\mu g\ m^{-3}$ against an active denuder reference[45], other authors have found them to substantially overestimate $NH_3$ concentrations at ambient levels[1,46]. In the U.K. during the 1980s and 1990s, an empirical correction factor of $\times 0.45$ was applied to diffusion tube results for national mapping of monitoring data[1,11], although it was acknowledged that this correction factor was very uncertain. The cause of the overestimation was not certain, with possible explanations including wind enhancement, sampling of aerosol $NH_4^+$ by open tubes, or migration of collected $NH_4^+$ along tube walls[42].

More recent tests have similarly shown variable results. Wyers et al.[43] tested diffusion tubes at relatively high concentrations (2 to 20 µg $m^{-3}$) in the Netherlands and found wind enhancement could lead to up to 19% overestimation in open tubes, but that otherwise the tubes had good agreement with active sampling. A similar agreement using 3.5-cm membrane diffusion tubes was found by Thijsse et al.[28] for concentrations mostly from the Netherlands in similar conditions, and these authors concluded that the method was reliable for monthly sampling. However, it should be noted that the data of Thijsse et al.[28] suggests an overestimation at low concentrations, with an intercept of 0.5 to 1 µg $m^{-3}$ in the regression.

This finding is supported by extensive analysis of $NH_3$ concentrations in the U.K., where 3.5-cm diffusion tubes have been compared against active denuder sampling[47]. Sutton et al.[13] also showed that replacing the membrane after sampling had an effect, reducing the intercept from around 1.5 to 0.8 µg $m^{-3}$. Lower blanks and detection limits were also achieved by capping the membrane inlet with an extra protective cap prior to sampling[13].

Fig. 4 shows a comparison of 3.5-cm membrane diffusion tubes against denuder sampling conducted by the Centre for Ecology and Hydrology (CEH). This indicates an intercept of 0.4 µg $m^{-3}$, with a slope of slightly less than 1. The less than unity slope may be attributed to the extra diffusion resistance of the membrane, whereas the nonzero intercept is probably due to the difference between measured and actual ‘blanks’ (Eq. 2).

The data of Thijsse et al.[28] and those presented in Fig. 4 show that diffusion tubes can measure concentrations >1 µg $m^{-3}$ to obtain annual means, but must be calibrated against a reference. The fact that different workers obtain varying relationships to active sampling, even for the same diffusion tube design, underlies the importance of establishing a calibration against a reference in applying diffusion tubes for $NH_3$[44].

The conclusion is that although $NH_3$ diffusion tubes can be shown to perform adequately, any implementation must be supported by ongoing reference data by active sampling across the full range of concentrations encountered. Indeed it may be noted that this was the purpose for which the CEH data shown in Fig. 4 were collected.

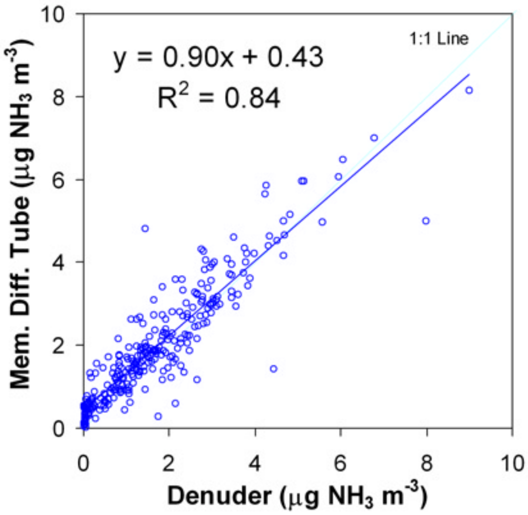

**FIGURE 4.** Comparison of individual monthly $NH_3$ concentrations from triplicate membrane diffusion tubes operated alongside an active denuder system, as part of the ongoing methods validation at nine sites in the DETR U.K. National Ammonia Monitoring Network.

### Performance of Badge Samplers for $NH_3$

Badge-type samplers have sampling rates that are 10 to 200 times greater than diffusion tubes (Table 1). The most sensitive sampler is the Willems badge developed by Willems and Hofschreuder, although it can become saturated very quickly at high concentrations or if used for longer-term sampling[43]. The Willems badge was used to map $NH_3$ concentration across Southern Ireland in the late 1990s[48]. Monthly sampling would have been adequate to provide the temporal and spatial details required, but weekly sampling was used to avoid saturation problems with the sampler, which demanded greater effort and resources. The Willems badge is also sensitive to the effect of wind speed (see "Effects of Wind Speed on Passive Sampling"), which has to be corrected for in the derivation of an effective sampling rate for the sampler[7,21]. The Ferm badge[15] samples at a lower rate than the Willems badge and would therefore be suitable for long-term sampling over a wider $NH_3$ concentration range. However, the design of the sampler means that the manipulation of the sampler is difficult in the field while the top cap can also come off, accidentally exposing the coated filter inside during handling.

**TABLE 2**
**Comparison of Atmospheric $NH_3$ Concentrations from Different Laboratories**

| | Measured Air $NH_3$ Concentration (µg $m^{-3}$) | |
|---|---|---|
| | Cheshire (8 sites) | East Anglia (14 sites) |
| Independent Laboratories | 20 - 40 | 0 -1000 |
| CEH Edinburgh | 2 - 4 | 0.5 - 20 |

*Note:* Measurements were made using the same type of membrane diffusion tubes.

### Contamination/Operator-Specific Error

$NH_3$ is ubiquitous in the environment and high concentrations are often encountered in the laboratory environment, with concentrations of up to 60 µg $NH_3$ $m^{-3}$ being measured (CEH, unpublished data). $NH_3$ is readily adsorbed to surfaces and $NH_3$ samplers are easily contaminated, either through contamination with laboratory air if proper care is not exercised, or through ammonium on the hands and fingers during handling and through $NH_3$ present in human breath[10]. Therefore, substantial errors in $NH_3$ sampling with passive samplers can occur if a proper protocol of cleaning, preparation, exposure, and storage of samplers is not strictly adhered to. For this reason, there is serious concern that errors associated with $NH_3$ passive samplers are partly operator specific.

The derivation of a sound sampling protocol and the rigorous testing and validation of a passive sampling methodology against a reference active sampling method is essential to ensure that the passive samplers give accurate and reliable air measurements. Problems arise when companies with little or no experience in the operation of passive diffusion samplers provide an air monitoring service using commercially available diffusion samplers. The manufacturers, in turn, are often unable to provide any details of sampling protocol or proper use of the samplers. A comparison of measurements by independent laboratories and CEH highlights the problem very clearly (Table 2).

The discrepancy in $NH_3$ data in the Cheshire study, which varied by a factor of 10, suggests a systematic error in the analysis and/or calculation of air $NH_3$ concentrations by the laboratory. The random concentrations of 0 to 1,000 µg $NH_3$ $m^{-3}$ reported by a second company in the East Anglia study, however, point to serious problems in the entire measurement process. The laboratory in this instance used single samplers for determination at each sampling location. Good practice requires replicate samplers to be exposed in parallel to test precision and to identify any potential contamination problems for every sampling period.

A number of important factors in the preparation and subsequent treatment of exposed $NH_3$ passive samplers have been identified, which produced more precise and accurate measurement data. This includes the replacement of exposed membranes immediately after exposure (see "Passive Sampling of $NH_3$" section), a precaution also supported by Ferm and Rodhe[39] for the Ferm sampler. We have also found that the standard procedure where the membrane inlet is not covered up after preparation gives high and variable blanks, despite being stored inside polystyrene containers, which in turn are stored inside grip-seal bags. After 1 week of storage following preparation, the coated grids of uncovered membrane diffusion tubes were found to be contaminated with 0.25 ± 0.06 µg $NH_4^+$ (n = 12), compared with 0.05 ± 0.01 µg $NH_4^+$ (n = 18) from diffusion tubes where the membrane inlets were covered with snug-fitting caps. The caps were either sourced (diffusion tubes) or made specially (ALPHA [Adapted Low Cost Passive High Absorption] samplers) to fit over the membrane inlet. This ensured that sampling does not begin until the cap is removed.

## DISCUSSION

### Recommendations for $NO_2$ and $NH_3$ Sampling

Based on the above review, it is clear that passive samplers can be used with some success to monitor ambient levels of $NO_2$ and $NH_3$, but that there are also a number of uncertainties and pitfalls which need to be considered. With regard to the application of passive samplers for ambient monitoring of $NO_2$ and $NH_3$ on a weekly or monthly timescales, the following recommendations can be made:

1. Passive diffusion samplers should be tested and validated against reference active sampling methods to ensure the reliability of data and where applicable, to obtain correction functions that can be used to correct the measured $NO_2$ and $NH_3$ concentration from the samplers using the theoretical diffusion rates. Tests should be carried out according to expected environmental conditions, anticipated pollutant concentration range, and exposure periods required.
2. Replicate samplers should be exposed in parallel to provide estimates of precision of the method and to identify contamination artefacts.
3. Written protocols, including QC/QA procedures, should be established giving clear guidelines on how to prepare, handle, expose, and analyse the passive samplers and how to calculate air concentration.
4. To minimise wind effects, where open-type diffusion tubes are used, they should be located away from the edges of buildings where there are likely to be large updrafts. In exposed sites, wind shelters should be used to reduce wind turbulence and to protect the samplers from rain.
5. Sampler types should be matched to measurement requirements. Badge-type samplers can be used to monitor low concentrations of $NO_2$ and $NH_3$ in background areas, but may not be suitable for monitoring in source regions or for long-term exposures (1 month or more) due to potential saturation problems. Diffusion tubes are low-sensitivity samplers and measurements for $NH_3$ are rather uncertain at background areas with concentrations below 2 µg $NH_3$ $m^{-3}$ (see "Uncertainties in the Reported Application of $NH_3$ Diffusion

Tubes"). However, $NH_3$ diffusion tubes are useful for providing annual mean estimates and for assessing spatial variability in source areas.

6. For $NO_2$ sampling, the sampler body should not transmit UV light that can photolyse $NO_2$. Acrylic is a suitable material[35], whereas quartz[32] and transparent polythene[18] are not. Badge-type samplers may also be preferred over tube-type samplers for $NO_2$ in urban and roadside locations to reduce interference from in-tube chemical reaction. Where there is uncertainty with regard to exposure-dependent loss of measured $NO_2$ (e.g., use of diffusion tubes with TEA coating), then short exposure periods (weekly) should be adopted. Collocation with chemiluminescence analyzers at key locations is also necessary to assess both positive bias due to chemical reaction and negative bias, which is related to sampler exposure duration.

7. For $NH_3$ sampling, contamination is a major problem and wearing disposable gloves in the handling of the samplers at all stages is recommended. The inlet of all samplers should be sealed off immediately after preparation to ensure that sampling does not begin until the inlet is exposed. After exposure, the membranes should be removed and the sampler should be sealed with a fresh, clean cap.

### Application of a Modified Passive Sampler for $NH_3$

From the considerations and recommendations above, a modified passive sampler for $NH_3$ has been developed. The new CEH ALPHA sampler (Fig. 5) was designed with an optimised sampling rate for long-term sampling (1 or 2 monthly periods) and was sensitive enough to resolve low concentrations (<1 µg $m^{-3}$ $NH_3$ and <0.5 ppb $NO_2$) in background areas. The membrane inlet is placed directly at the mouth of the sampler, so that the formation of a boundary layer in front of the membrane is minimised and a stable, turbulent-free diffusion path length is achieved behind the membrane. To make handling easier, the sampler body was extended behind the coated filter.

Results from an intercomparison between the ALPHA samplers and an active denuder (CEH DELTA) system[47] at Monte Bondone in northern Italy are shown in Fig. 6. Even with a 9-month average concentration of around 0.2 µg $m^{-3}$, the mean estimates agreed within 5%, while the results show a very close agreement of individual monthly results.

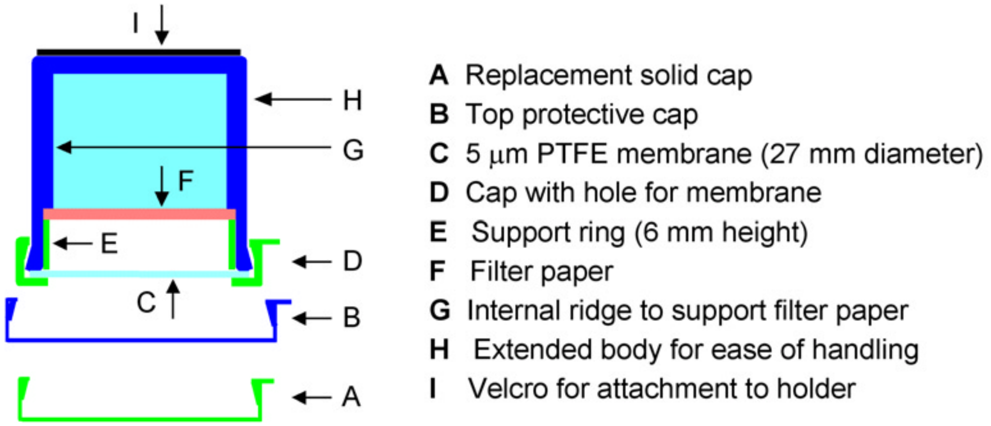

**FIGURE 5**. The new CEH ALPHA sampler.

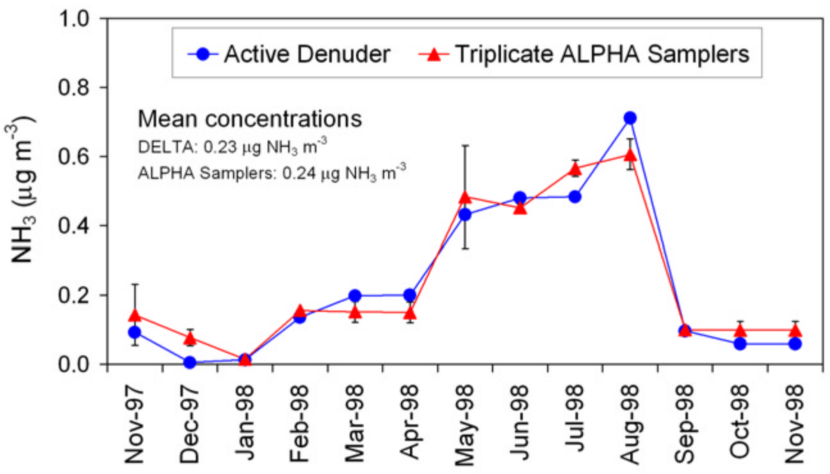

**FIGURE 6**. Intercomparison of $NH_3$ measurement results by ALPHA sampler and active denuder system at Monte Bondone, Italy (ECOMONT project). Each data point for the ALPHA sampler results represent the mean ± SD of replicate samples (n=3).

Membrane diffusion tubes, and more recently, the new ALPHA samplers, have been used at key locations in the U.K. National Ammonia Monitoring Network[13]. The Monitoring Network was set up in 1995 and is operated by CEH (formerly ITE) on behalf of the Department of Environment, Transport and the Regions (DETR). There are currently 90 sites being operated across the U.K. At 59 of these sites, an active sampling methodology using the CEH DELTA system[47] is used to provide the spatial and temporal patterns of $NH_3$ across the U.K., whereas ALPHA samplers are used to assess regional and local scale variability in air $NH_3$ concentrations in source regions. To provide an ongoing validation of the ALPHA sampler, the performance of the new ALPHA Samplers is continuously assessed at 9 U.K. sites within the network, and has also been tested within the EC ECOMONT project at several U.K. and European sites[49] against the CEH DELTA system. The comparison shown in Fig. 7 demonstrates an order of magnitude better precision with the ALPHA samplers compared with the diffusion tubes (Fig. 5), while the intercept is also proportionately smaller (0.05 μg $NH_3$ $m^{-3}$). The nonunity slope may again be attributed to the additional resistance due to the membrane and boundary layer. This regression is used to calibrate the ALPHA sampler data, which are mapped together with the data for active denuder sampling.

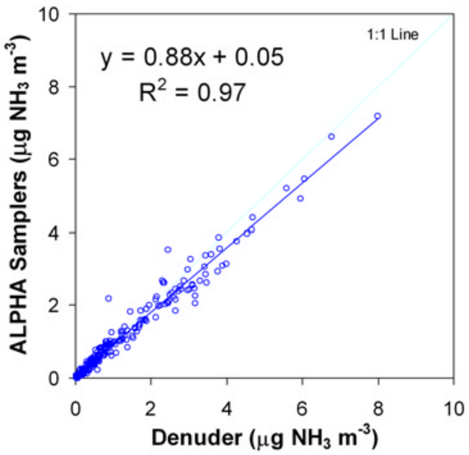

**FIGURE 7**. Comparison of individual monthly $NH_3$ concentrations from triplicate ALPHA samplers operated alongside an active denuder system, as part of the ongoing methods validation at 9 sites in the DETR U.K. National Ammonia Monitoring Network.

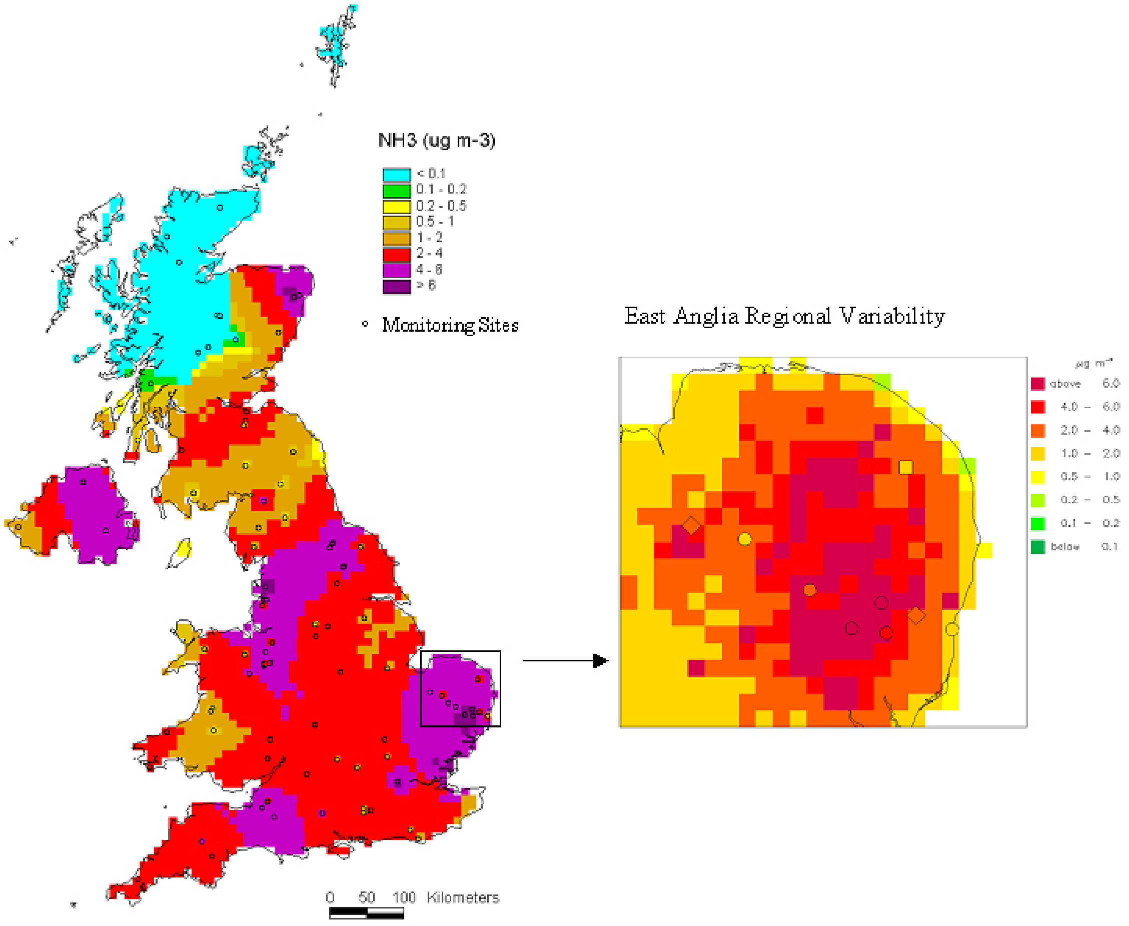

**FIGURE 8**. Interpolated atmospheric $NH_3$ concentrations from the U.K. National $NH_3$ Monitoring network and map of East Anglia showing (as colored points) the $NH_3$ concentrations determined by the monitoring network on a background of the FRAME predicted concentrations[50].

The results of this mapping at a U.K. and local scale are shown in Fig. 8. Data from the network confirm the high spatial variability in $NH_3$ concentration across the country, reflecting the large regional variability in $NH_3$ emissions[13]. The regional study in East Anglia shows a gradient in concentrations along a transect (Fig. 8), in agreement with predicted concentrations from an atmospheric transport model[50]. Work is now currently in progress for $NO_2$ sampling.

### CONCLUSIONS

Passive diffusion samplers can be used successfully to monitor $NO_2$ and $NH_3$ concentrations, provided that the methods used have been rigorously tested, validated, and, where necessary, calibrated against recognised reference methods. For $NO_2$ sampling, the open diffusion tube used in the U.K. is affected by wind speed, and care must be exercised in the selection of sampling locations. Positive bias due to chemical reaction is difficult to correct, but may be minimised by using badge- rather than tube-type samplers. Diffusion tube data from urban and curbside locations where chemical interference is most pronounced must therefore be treated with caution. On the other hand, negative bias reported for longer exposure times can lead to underestimation of $NO_2$ concentration, and more work is required to address the issue of sample stability. For $NH_3$ sampling, errors in passive sampling are often due to contamination artefacts. Clear guidelines on sampling protocol and careful handling of the samplers can reduce these errors, which are also minimised by an appropriate sampler architecture to match the concentration sensitivity required. Using the identified recommendations on sampler design, a new passive sampler for $NH_3$ and $NO_2$ has been developed and applied at U.K. and European sites.

### ACKNOWLEDGEMENTS

We are grateful to the Department of Environment, Transport and the Regions (DETR) for the funding of the U.K. National Ammonia Monitoring Network, the EU for the funding of the EC ECOMONT project, and for supporting funding from CEH Programmes. We would also like to thank the local site operators in the U.K. network and the researchers within the EC ECOMONT project, without whom the monitoring activity would not have been possible.
